# *N*-(4-Methyl­phen­yl)benzene­sulfonamide

**DOI:** 10.1107/S1600536810002278

**Published:** 2010-01-23

**Authors:** B. Thimme Gowda, Sabine Foro, P. G. Nirmala, Hartmut Fuess

**Affiliations:** a Department of Chemistry, Mangalore University, Mangalagangotri 574 199, Mangalore, India; b Institute of Materials Science, Darmstadt University of Technology, Petersenstrasse 23, D-64287 Darmstadt, Germany

## Abstract

The asymmetric unit of the title compound, C_13_H_13_NO_2_S, contains two independent mol­ecules. The dihedral angles between the aromatic rings in the two mol­ecules are 78.0 (1) and 74.0 (1)°. In the crystal, inter­molecular N—H⋯O hydrogen bonds pack the mol­ecules into a three-dimensional structure.

## Related literature

For the preparation of the title compound, see: Gowda *et al.* (2005 ▶). For related structures, see: Gelbrich *et al.* (2007 ▶); Gowda *et al.* (2008 ▶, 2010 ▶); Perlovich *et al.* (2006 ▶).
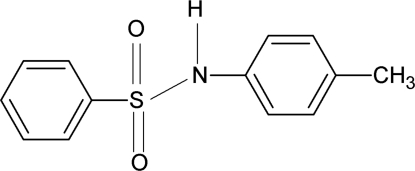


## Experimental

### 

#### Crystal data

C_13_H_13_NO_2_S
*M* *_r_* = 247.30
Monoclinic, 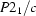

*a* = 10.8963 (7) Å
*b* = 9.6981 (7) Å
*c* = 24.089 (2) Å
β = 101.335 (6)°
*V* = 2495.9 (3) Å^3^
*Z* = 8
Mo *K*α radiation
μ = 0.25 mm^−1^
*T* = 299 K
0.48 × 0.36 × 0.36 mm

#### Data collection

Oxford Diffraction Xcalibur diffractometer with a Sapphire CCD detector
Absorption correction: multi-scan (*CrysAlis RED*; Oxford Diffraction, 2009 ▶) *T*_min_ = 0.890, *T*_max_ = 0.916
11057 measured reflections
5102 independent reflections
3835 reflections with *I* > 2σ(*I*)
*R*_int_ = 0.016

#### Refinement

*R*[*F* ^2^ > 2σ(*F* ^2^)] = 0.040
*wR*(*F* ^2^) = 0.115
*S* = 1.07
5102 reflections
317 parameters
2 restraints
H atoms treated by a mixture of independent and constrained refinement
Δρ_max_ = 0.25 e Å^−3^
Δρ_min_ = −0.44 e Å^−3^

### 

Data collection: *CrysAlis CCD* (Oxford Diffraction, 2009 ▶); cell refinement: *CrysAlis RED* (Oxford Diffraction, 2009 ▶); data reduction: *CrysAlis RED*; program(s) used to solve structure: *SHELXS97* (Sheldrick, 2008 ▶); program(s) used to refine structure: *SHELXL97* (Sheldrick, 2008 ▶); molecular graphics: *PLATON* (Spek, 2009 ▶); software used to prepare material for publication: *SHELXL97*.

## Supplementary Material

Crystal structure: contains datablocks I, global. DOI: 10.1107/S1600536810002278/bt5173sup1.cif

Structure factors: contains datablocks I. DOI: 10.1107/S1600536810002278/bt5173Isup2.hkl

Additional supplementary materials:  crystallographic information; 3D view; checkCIF report

## Figures and Tables

**Table 1:** Hydrogen-bond geometry (Å, °)

*D*—H⋯*A*	*D*—H	H⋯*A*	*D*⋯*A*	*D*—H⋯*A*
N1—H1*N*⋯O2^i^	0.86 (1)	2.09 (1)	2.924 (2)	166 (2)
N2—H2*N*⋯O1	0.85 (1)	2.17 (1)	3.0056 (19)	168 (2)

Symmetry code: (i) 
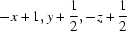
.

**Fig. 1.**
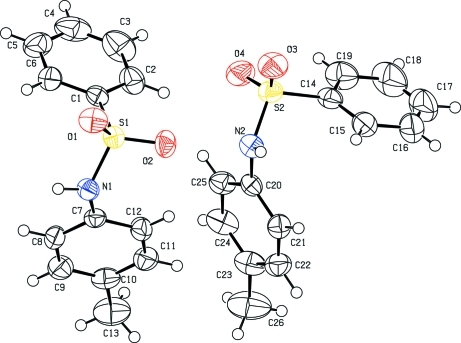
Molecular structure of the title compound, showing the atom labelling scheme and displacement ellipsoids drawn at the 50% probability level.

**Fig. 2.**
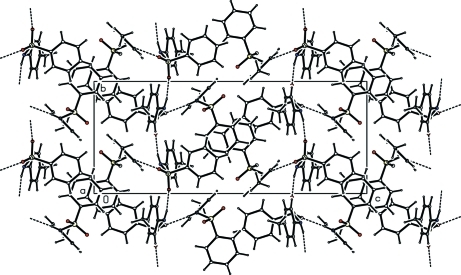
Molecular packing of the title compound with hydrogen bonding shown as dashed lines.

## supplementary crystallographic information

### Comment

As a part of studying the effect of substituents on the structures of *N*-(aryl)arylsulfonamides (Gowda *et al.*, 2008; 2010), the crystal structure of *N*-(4-methylphenyl)benzenesulfonamide   has been determined. The asymmetric unit   contains two independent molecules (Fig. 1).

The conformations of the N—C bonds in the C—SO_2_—NH—C segments of both molecules   have *gauche* torsions with respect to the S═O bonds. The molecules are bent at the *S* atoms with the C1—SO_2_—NH—C7 and C14—SO_2_—NH—C20 torsion angles of 59.5 (2)° and -55.2 (2)°, respectively, in the 2 molecules.

The two aromatic rings   are tilted relative to each other by 78.0 (1)° in molecule 1 and 74.0 (1)° in molecule 2, compared to the values of 61.5 (1)° in *N*-(2-methylphenyl)benzenesulfonamide (II) (Gowda *et al.*, 2008) and 67.9 (1)° (molecule 1) and 68.6 (1)° (molecule 2) in *N*-(3-methylphenyl)benzenesulfonamide (III) (Gowda *et al.*, 2010)

The other bond parameters are similar to those observed in (II), (III) and other aryl sulfonamides (Perlovich *et al.*, 2006; Gelbrich *et al.*, 2007). The crystal packing stabilized by intermolecular N—H···O hydrogen bonds (Table 1) is shown in Fig.2.

### Experimental

The solution of benzene (10 ml) in chloroform (40 ml) was treated dropwise with chlorosulfonic acid (25 ml) at 0 ° C. After the initial evolution of hydrogen chloride subsided, the reaction mixture was brought to room temperature and poured into crushed ice in a beaker. The chloroform layer was separated, washed with cold water and allowed to evaporate slowly. The residual benzenesulfonylchloride was treated with *p*-toluidine in the stoichiometric ratio and boiled for ten minutes. The reaction mixture was then cooled to room temperature and added to ice cold water (100 ml). The resultant solid *N*-(4-methylphenyl)benzenesulfonamide was filtered under suction and washed thoroughly with cold water. It was then recrystallized to constant melting point from dilute ethanol. The purity of the compound was checked and characterized by recording its infrared and NMR spectra (Gowda *et al.*, 2005).

The single crystals used in X-ray diffraction studies were grown in ethanolic solution by a slow evaporation at room temperature.

### Refinement

The H atoms of the NH groups were located in a difference map and refined with the N—H distance restrained to  0.86 (1) Å.  H atoms bonded to C were positioned with idealized geometry using a riding model with C—H = 0.93–0.96 Å and  with their isotropic displacement parameters  set to 1.2 times of the *U*_eq_ of the parent C atom.

### Crystal data

**Table:**

C_13_H_13_NO_2_S	*F*(000) = 1040
*M**_r_* = 247.30	*D*_x_ = 1.316 Mg m^−^^3^
Monoclinic, *P*2_1_/*c*	Mo *K*α radiation, λ = 0.71073 Å
Hall symbol: -P 2ybc	Cell parameters from 3814 reflections
*a* = 10.8963 (7) Å	θ = 2.6–27.8°
*b* = 9.6981 (7) Å	µ = 0.25 mm^−^^1^
*c* = 24.089 (2) Å	*T* = 299 K
β = 101.335 (6)°	Prism, colourless
*V* = 2495.9 (3) Å^3^	0.48 × 0.36 × 0.36 mm
*Z* = 8	

### Data collection

**Table:**

Oxford Diffraction Xcalibur diffractometer with a Sapphire CCD detector	5102 independent reflections
Radiation source: fine-focus sealed tube	3835 reflections with *I* > 2σ(*I*)
graphite	*R*_int_ = 0.016
Rotation method data acquisition using ω and φ scans	θ_max_ = 26.4°, θ_min_ = 2.7°
Absorption correction: multi-scan (*CrysAlis RED*; Oxford Diffraction, 2009)	*h* = −13→6
*T*_min_ = 0.890, *T*_max_ = 0.916	*k* = −8→12
11057 measured reflections	*l* = −30→29

### Refinement

**Table:**

Refinement on *F*^2^	Primary atom site location: structure-invariant direct methods
Least-squares matrix: full	Secondary atom site location: difference Fourier map
*R*[*F*^2^ > 2σ(*F*^2^)] = 0.040	Hydrogen site location: inferred from neighbouring sites
*wR*(*F*^2^) = 0.115	H atoms treated by a mixture of independent and constrained refinement
*S* = 1.07	*w* = 1/[σ^2^(*F*_o_^2^) + (0.0678*P*)^2^ + 0.1533*P*] where *P* = (*F*_o_^2^ + 2*F*_c_^2^)/3
5102 reflections	(Δ/σ)_max_ = 0.022
317 parameters	Δρ_max_ = 0.25 e Å^−^^3^
2 restraints	Δρ_min_ = −0.44 e Å^−^^3^

### Special details

**Table:**

Experimental. CrysAlis RED (Oxford Diffraction, 2009) Empirical absorption correction using spherical harmonics, implemented in SCALE3 ABSPACK scaling algorithm.
Geometry. All e.s.d.'s (except the e.s.d. in the dihedral angle between two l.s. planes) are estimated using the full covariance matrix. The cell e.s.d.'s are taken into account individually in the estimation of e.s.d.'s in distances, angles and torsion angles; correlations between e.s.d.'s in cell parameters are only used when they are defined by crystal symmetry. An approximate (isotropic) treatment of cell e.s.d.'s is used for estimating e.s.d.'s involving l.s. planes.
Refinement. Refinement of *F*^2^ against ALL reflections. The weighted *R*-factor *wR* and goodness of fit *S* are based on *F*^2^, conventional *R*-factors *R* are based on *F*, with *F* set to zero for negative *F*^2^. The threshold expression of *F*^2^ > σ(*F*^2^) is used only for calculating *R*-factors(gt) *etc*. and is not relevant to the choice of reflections for refinement. *R*-factors based on *F*^2^ are statistically about twice as large as those based on *F*, and *R*- factors based on ALL data will be even larger.

### Fractional atomic coordinates and isotropic or equivalent isotropic displacement parameters (Å^2^)

**Table:**

	*x*	*y*	*z*	*U*_iso_*/*U*_eq_	
C1	0.49646 (15)	0.23424 (19)	0.34166 (7)	0.0352 (4)	
C2	0.54145 (19)	0.1458 (2)	0.38595 (8)	0.0540 (5)	
H2	0.5706	0.0585	0.3792	0.065*	
C3	0.5422 (2)	0.1902 (3)	0.44079 (9)	0.0743 (7)	
H3	0.5714	0.1317	0.4711	0.089*	
C4	0.5009 (2)	0.3177 (3)	0.45059 (10)	0.0729 (7)	
H4	0.5017	0.3455	0.4876	0.087*	
C5	0.4576 (2)	0.4073 (3)	0.40631 (10)	0.0637 (6)	
H5	0.4304	0.4952	0.4135	0.076*	
C6	0.45504 (17)	0.3650 (2)	0.35136 (8)	0.0473 (5)	
H6	0.4257	0.4239	0.3212	0.057*	
C7	0.72265 (15)	0.26027 (18)	0.26558 (7)	0.0345 (4)	
C8	0.78605 (17)	0.38435 (19)	0.27057 (8)	0.0437 (4)	
H8	0.7424	0.4664	0.2618	0.052*	
C9	0.91509 (18)	0.3864 (2)	0.28866 (9)	0.0519 (5)	
H9	0.9569	0.4705	0.2916	0.062*	
C10	0.98274 (17)	0.2673 (2)	0.30237 (8)	0.0483 (5)	
C11	0.91807 (17)	0.1439 (2)	0.29706 (8)	0.0492 (5)	
H11	0.9621	0.0621	0.3061	0.059*	
C12	0.78924 (17)	0.1386 (2)	0.27865 (8)	0.0445 (4)	
H12	0.7478	0.0543	0.2751	0.053*	
C13	1.1240 (2)	0.2704 (3)	0.32199 (12)	0.0782 (8)	
H13A	1.1543	0.3620	0.3178	0.094*	
H13B	1.1619	0.2076	0.2995	0.094*	
H13C	1.1452	0.2435	0.3611	0.094*	
N1	0.59074 (13)	0.26099 (17)	0.24393 (6)	0.0380 (4)	
H1N	0.5641 (19)	0.3419 (13)	0.2338 (9)	0.061 (7)*	
O1	0.36880 (11)	0.21729 (15)	0.23898 (5)	0.0483 (3)	
O2	0.52235 (12)	0.03473 (13)	0.27441 (6)	0.0484 (3)	
S1	0.48854 (4)	0.17773 (5)	0.271806 (17)	0.03439 (13)	
C14	0.24474 (16)	0.1155 (2)	0.04914 (8)	0.0433 (4)	
C15	0.23588 (19)	0.0133 (2)	0.08819 (10)	0.0539 (5)	
H15	0.2661	0.0292	0.1265	0.065*	
C16	0.1824 (2)	−0.1121 (3)	0.07043 (13)	0.0746 (7)	
H16	0.1760	−0.1812	0.0965	0.089*	
C17	0.1387 (3)	−0.1331 (3)	0.01356 (16)	0.0934 (10)	
H17	0.1026	−0.2175	0.0013	0.112*	
C18	0.1469 (3)	−0.0333 (4)	−0.02519 (13)	0.0927 (10)	
H18	0.1169	−0.0504	−0.0634	0.111*	
C19	0.1998 (2)	0.0942 (3)	−0.00817 (9)	0.0691 (7)	
H19	0.2050	0.1631	−0.0345	0.083*	
C20	0.12459 (15)	0.34730 (18)	0.11770 (7)	0.0355 (4)	
C21	0.05945 (18)	0.2725 (2)	0.15145 (8)	0.0440 (4)	
H21	0.1015	0.2087	0.1772	0.053*	
C22	−0.06751 (19)	0.2922 (2)	0.14710 (9)	0.0521 (5)	
H22	−0.1096	0.2421	0.1704	0.063*	
C23	−0.13356 (18)	0.3848 (3)	0.10883 (8)	0.0544 (6)	
C24	−0.06742 (18)	0.4595 (2)	0.07557 (8)	0.0558 (6)	
H24	−0.1099	0.5226	0.0497	0.067*	
C25	0.06092 (18)	0.4430 (2)	0.07982 (7)	0.0469 (5)	
H25	0.1037	0.4955	0.0575	0.056*	
C26	−0.2728 (2)	0.4058 (4)	0.10399 (11)	0.0918 (10)	
H26A	−0.2892	0.4368	0.1397	0.110*	
H26B	−0.3157	0.3203	0.0938	0.110*	
H26C	−0.3019	0.4738	0.0755	0.110*	
N2	0.25663 (14)	0.32896 (17)	0.12379 (6)	0.0404 (4)	
H2N	0.2978 (16)	0.2947 (19)	0.1543 (6)	0.047 (6)*	
O3	0.44699 (12)	0.24737 (16)	0.09618 (7)	0.0607 (4)	
O4	0.28964 (14)	0.36865 (16)	0.02590 (6)	0.0614 (4)	
S2	0.31882 (4)	0.27343 (5)	0.07160 (2)	0.04299 (15)	

### Atomic displacement parameters (Å^2^)

**Table:**

	*U*^11^	*U*^22^	*U*^33^	*U*^12^	*U*^13^	*U*^23^
C1	0.0322 (9)	0.0384 (10)	0.0348 (9)	−0.0071 (7)	0.0058 (7)	0.0000 (7)
C2	0.0650 (13)	0.0520 (13)	0.0431 (11)	−0.0009 (10)	0.0057 (9)	0.0080 (9)
C3	0.0945 (19)	0.087 (2)	0.0382 (12)	−0.0135 (15)	0.0046 (12)	0.0091 (12)
C4	0.0800 (17)	0.099 (2)	0.0450 (12)	−0.0294 (15)	0.0256 (12)	−0.0179 (14)
C5	0.0673 (14)	0.0626 (15)	0.0674 (15)	−0.0120 (11)	0.0287 (12)	−0.0233 (13)
C6	0.0494 (11)	0.0438 (12)	0.0506 (11)	−0.0020 (9)	0.0143 (9)	−0.0026 (9)
C7	0.0341 (9)	0.0401 (10)	0.0308 (8)	0.0032 (7)	0.0098 (7)	0.0025 (7)
C8	0.0412 (10)	0.0343 (10)	0.0576 (11)	0.0038 (8)	0.0142 (8)	−0.0011 (9)
C9	0.0435 (11)	0.0485 (13)	0.0661 (13)	−0.0092 (9)	0.0169 (9)	−0.0090 (10)
C10	0.0345 (10)	0.0672 (14)	0.0444 (11)	0.0020 (9)	0.0107 (8)	0.0004 (10)
C11	0.0393 (10)	0.0540 (13)	0.0551 (12)	0.0137 (9)	0.0116 (9)	0.0133 (10)
C12	0.0402 (10)	0.0388 (10)	0.0551 (11)	0.0037 (8)	0.0110 (8)	0.0081 (9)
C13	0.0382 (12)	0.110 (2)	0.0846 (18)	−0.0009 (13)	0.0089 (11)	−0.0004 (16)
N1	0.0324 (8)	0.0403 (9)	0.0407 (8)	0.0033 (7)	0.0060 (6)	0.0086 (7)
O1	0.0315 (7)	0.0660 (9)	0.0430 (7)	−0.0024 (6)	−0.0033 (5)	0.0012 (6)
O2	0.0528 (8)	0.0319 (7)	0.0592 (8)	−0.0058 (6)	0.0080 (6)	−0.0083 (6)
S1	0.0319 (2)	0.0350 (3)	0.0343 (2)	−0.00365 (18)	0.00181 (16)	−0.00231 (18)
C14	0.0343 (9)	0.0523 (12)	0.0441 (10)	0.0007 (8)	0.0102 (8)	−0.0094 (9)
C15	0.0524 (12)	0.0514 (13)	0.0601 (13)	−0.0001 (10)	0.0164 (10)	−0.0089 (10)
C16	0.0732 (16)	0.0505 (15)	0.105 (2)	−0.0068 (12)	0.0305 (15)	−0.0141 (14)
C17	0.084 (2)	0.076 (2)	0.126 (3)	−0.0238 (16)	0.0340 (19)	−0.051 (2)
C18	0.090 (2)	0.109 (3)	0.0741 (18)	−0.0173 (18)	0.0047 (15)	−0.0492 (18)
C19	0.0675 (15)	0.0919 (19)	0.0462 (12)	−0.0078 (13)	0.0070 (11)	−0.0188 (12)
C20	0.0378 (9)	0.0373 (10)	0.0299 (8)	0.0011 (7)	0.0030 (7)	−0.0072 (7)
C21	0.0495 (11)	0.0420 (11)	0.0397 (10)	−0.0007 (9)	0.0070 (8)	0.0009 (8)
C22	0.0523 (12)	0.0597 (14)	0.0473 (11)	−0.0102 (10)	0.0170 (9)	−0.0080 (10)
C23	0.0390 (10)	0.0812 (16)	0.0406 (10)	0.0004 (10)	0.0017 (8)	−0.0185 (10)
C24	0.0503 (12)	0.0785 (16)	0.0351 (10)	0.0199 (11)	−0.0003 (9)	0.0012 (10)
C25	0.0512 (11)	0.0550 (13)	0.0353 (9)	0.0064 (9)	0.0105 (8)	0.0042 (9)
C26	0.0420 (13)	0.156 (3)	0.0749 (17)	0.0045 (15)	0.0056 (12)	−0.0146 (18)
N2	0.0392 (8)	0.0470 (10)	0.0324 (8)	0.0027 (7)	0.0007 (6)	−0.0040 (7)
O3	0.0317 (7)	0.0740 (11)	0.0751 (10)	−0.0028 (6)	0.0074 (7)	−0.0085 (8)
O4	0.0740 (10)	0.0628 (10)	0.0528 (8)	0.0055 (8)	0.0254 (7)	0.0143 (7)
S2	0.0366 (3)	0.0491 (3)	0.0442 (3)	−0.0012 (2)	0.01009 (19)	−0.0016 (2)

### Geometric parameters (Å, °)

**Table:**

C1—C6	1.381 (3)	C14—C15	1.383 (3)
C1—C2	1.382 (2)	C14—C19	1.386 (3)
C1—S1	1.7556 (17)	C14—S2	1.766 (2)
C2—C3	1.388 (3)	C15—C16	1.380 (3)
C2—H2	0.9300	C15—H15	0.9300
C3—C4	1.352 (4)	C16—C17	1.375 (4)
C3—H3	0.9300	C16—H16	0.9300
C4—C5	1.385 (3)	C17—C18	1.360 (4)
C4—H4	0.9300	C17—H17	0.9300
C5—C6	1.381 (3)	C18—C19	1.391 (4)
C5—H5	0.9300	C18—H18	0.9300
C6—H6	0.9300	C19—H19	0.9300
C7—C8	1.381 (3)	C20—C21	1.385 (3)
C7—C12	1.389 (2)	C20—C25	1.388 (2)
C7—N1	1.429 (2)	C20—N2	1.428 (2)
C8—C9	1.388 (2)	C21—C22	1.380 (3)
C8—H8	0.9300	C21—H21	0.9300
C9—C10	1.375 (3)	C22—C23	1.383 (3)
C9—H9	0.9300	C22—H22	0.9300
C10—C11	1.382 (3)	C23—C24	1.383 (3)
C10—C13	1.519 (3)	C23—C26	1.512 (3)
C11—C12	1.388 (2)	C24—C25	1.391 (3)
C11—H11	0.9300	C24—H24	0.9300
C12—H12	0.9300	C25—H25	0.9300
C13—H13A	0.9600	C26—H26A	0.9600
C13—H13B	0.9600	C26—H26B	0.9600
C13—H13C	0.9600	C26—H26C	0.9600
N1—S1	1.6239 (16)	N2—S2	1.6325 (15)
N1—H1N	0.855 (9)	N2—H2N	0.850 (9)
O1—S1	1.4384 (12)	O3—S2	1.4282 (13)
O2—S1	1.4331 (13)	O4—S2	1.4240 (14)
			
C6—C1—C2	121.15 (18)	C15—C14—C19	120.8 (2)
C6—C1—S1	119.52 (14)	C15—C14—S2	120.13 (15)
C2—C1—S1	119.31 (15)	C19—C14—S2	119.01 (18)
C1—C2—C3	118.5 (2)	C16—C15—C14	120.2 (2)
C1—C2—H2	120.7	C16—C15—H15	119.9
C3—C2—H2	120.7	C14—C15—H15	119.9
C4—C3—C2	120.6 (2)	C17—C16—C15	118.8 (3)
C4—C3—H3	119.7	C17—C16—H16	120.6
C2—C3—H3	119.7	C15—C16—H16	120.6
C3—C4—C5	121.0 (2)	C18—C17—C16	121.4 (3)
C3—C4—H4	119.5	C18—C17—H17	119.3
C5—C4—H4	119.5	C16—C17—H17	119.3
C6—C5—C4	119.4 (2)	C17—C18—C19	120.6 (3)
C6—C5—H5	120.3	C17—C18—H18	119.7
C4—C5—H5	120.3	C19—C18—H18	119.7
C1—C6—C5	119.3 (2)	C14—C19—C18	118.1 (3)
C1—C6—H6	120.3	C14—C19—H19	121.0
C5—C6—H6	120.3	C18—C19—H19	121.0
C8—C7—C12	119.38 (16)	C21—C20—C25	119.24 (17)
C8—C7—N1	118.52 (15)	C21—C20—N2	119.78 (16)
C12—C7—N1	122.01 (16)	C25—C20—N2	120.94 (16)
C7—C8—C9	119.90 (17)	C22—C21—C20	120.32 (18)
C7—C8—H8	120.0	C22—C21—H21	119.8
C9—C8—H8	120.0	C20—C21—H21	119.8
C10—C9—C8	121.69 (19)	C21—C22—C23	121.54 (19)
C10—C9—H9	119.2	C21—C22—H22	119.2
C8—C9—H9	119.2	C23—C22—H22	119.2
C9—C10—C11	117.77 (17)	C22—C23—C24	117.65 (18)
C9—C10—C13	121.4 (2)	C22—C23—C26	121.4 (2)
C11—C10—C13	120.8 (2)	C24—C23—C26	120.9 (2)
C10—C11—C12	121.79 (18)	C23—C24—C25	121.82 (19)
C10—C11—H11	119.1	C23—C24—H24	119.1
C12—C11—H11	119.1	C25—C24—H24	119.1
C11—C12—C7	119.47 (18)	C20—C25—C24	119.41 (18)
C11—C12—H12	120.3	C20—C25—H25	120.3
C7—C12—H12	120.3	C24—C25—H25	120.3
C10—C13—H13A	109.5	C23—C26—H26A	109.5
C10—C13—H13B	109.5	C23—C26—H26B	109.5
H13A—C13—H13B	109.5	H26A—C26—H26B	109.5
C10—C13—H13C	109.5	C23—C26—H26C	109.5
H13A—C13—H13C	109.5	H26A—C26—H26C	109.5
H13B—C13—H13C	109.5	H26B—C26—H26C	109.5
C7—N1—S1	124.67 (12)	C20—N2—S2	121.62 (11)
C7—N1—H1N	111.7 (15)	C20—N2—H2N	119.2 (13)
S1—N1—H1N	110.6 (14)	S2—N2—H2N	107.9 (13)
O2—S1—O1	118.67 (8)	O4—S2—O3	119.13 (9)
O2—S1—N1	107.94 (8)	O4—S2—N2	108.54 (9)
O1—S1—N1	105.17 (8)	O3—S2—N2	105.02 (8)
O2—S1—C1	107.24 (8)	O4—S2—C14	107.85 (9)
O1—S1—C1	108.18 (8)	O3—S2—C14	108.93 (9)
N1—S1—C1	109.42 (8)	N2—S2—C14	106.74 (8)
			
C6—C1—C2—C3	1.1 (3)	C19—C14—C15—C16	0.3 (3)
S1—C1—C2—C3	−177.38 (17)	S2—C14—C15—C16	−177.47 (16)
C1—C2—C3—C4	−0.6 (4)	C14—C15—C16—C17	0.1 (3)
C2—C3—C4—C5	−0.4 (4)	C15—C16—C17—C18	−0.1 (4)
C3—C4—C5—C6	0.9 (4)	C16—C17—C18—C19	−0.3 (5)
C2—C1—C6—C5	−0.6 (3)	C15—C14—C19—C18	−0.6 (3)
S1—C1—C6—C5	177.87 (14)	S2—C14—C19—C18	177.13 (18)
C4—C5—C6—C1	−0.4 (3)	C17—C18—C19—C14	0.6 (4)
C12—C7—C8—C9	−0.2 (3)	C25—C20—C21—C22	0.5 (3)
N1—C7—C8—C9	−176.66 (16)	N2—C20—C21—C22	178.13 (16)
C7—C8—C9—C10	−0.5 (3)	C20—C21—C22—C23	0.9 (3)
C8—C9—C10—C11	0.6 (3)	C21—C22—C23—C24	−1.3 (3)
C8—C9—C10—C13	179.84 (19)	C21—C22—C23—C26	179.8 (2)
C9—C10—C11—C12	−0.1 (3)	C22—C23—C24—C25	0.3 (3)
C13—C10—C11—C12	−179.32 (19)	C26—C23—C24—C25	179.2 (2)
C10—C11—C12—C7	−0.5 (3)	C21—C20—C25—C24	−1.5 (3)
C8—C7—C12—C11	0.7 (3)	N2—C20—C25—C24	−179.07 (17)
N1—C7—C12—C11	177.04 (16)	C23—C24—C25—C20	1.1 (3)
C8—C7—N1—S1	−133.52 (16)	C21—C20—N2—S2	118.91 (17)
C12—C7—N1—S1	50.1 (2)	C25—C20—N2—S2	−63.5 (2)
C7—N1—S1—O2	−56.90 (16)	C20—N2—S2—O4	60.77 (16)
C7—N1—S1—O1	175.49 (14)	C20—N2—S2—O3	−170.79 (14)
C7—N1—S1—C1	59.49 (17)	C20—N2—S2—C14	−55.23 (16)
C6—C1—S1—O2	−172.89 (14)	C15—C14—S2—O4	−166.93 (15)
C2—C1—S1—O2	5.65 (17)	C19—C14—S2—O4	15.29 (19)
C6—C1—S1—O1	−43.79 (16)	C15—C14—S2—O3	62.44 (18)
C2—C1—S1—O1	134.75 (15)	C19—C14—S2—O3	−115.33 (17)
C6—C1—S1—N1	70.28 (16)	C15—C14—S2—N2	−50.46 (17)
C2—C1—S1—N1	−111.19 (16)	C19—C14—S2—N2	131.76 (17)

### Hydrogen-bond geometry (Å, °)

**Table:**

*D*—H···*A*	*D*—H	H···*A*	*D*···*A*	*D*—H···*A*
N1—H1N···O2^i^	0.86 (1)	2.09 (1)	2.924 (2)	166 (2)
N2—H2N···O1	0.85 (1)	2.17 (1)	3.0056 (19)	168 (2)

Symmetry codes: (i) −*x*+1, *y*+1/2, −*z*+1/2.
